# Absolute spectroscopy near 7.8 μm with a comb-locked extended-cavity quantum-cascade-laser

**DOI:** 10.1038/s41598-018-19188-2

**Published:** 2018-01-22

**Authors:** Marco Lamperti, Bidoor AlSaif, Davide Gatti, Martin Fermann, Paolo Laporta, Aamir Farooq, Marco Marangoni

**Affiliations:** 1 Physics Department of Politecnico di Milano and IFN-CNR, Via G. Previati 1/C, 23900 Lecco, Italy; 2 King Abdullah University of Science and Technology (KAUST), Clean Combustion Research Center (CCRC), Thuwal, 23955 Saudi Arabia; 3 IMRA America Inc., 1044 Woodridge Avenue, Ann Arbor, 48105 Michigan USA

## Abstract

We report for the first time the frequency locking of an extended-cavity quantum-cascade-laser (EC-QCL) to a near-infrared frequency comb. The locked laser source is exploited to carry out molecular spectroscopy around 7.8 μm with a line-centre frequency combined uncertainty of ~63 kHz. The strength of the approach, in view of an accurate retrieval of line centre frequencies over a spectral range as large as 100 cm^−1^, is demonstrated on the P(40), P(18) and R(31) lines of the fundamental rovibrational band of N_2_O covering the centre and edges of the P and R branches. The spectrometer has the potential to be straightforwardly extended to other spectral ranges, till 12 μm, which is the current wavelength limit for commercial cw EC-QCLs.

## Introduction

Since their invention, optical frequency combs have revitalized the field of precision molecular spectroscopy, making it possible to achieve accuracies at the kHz or even sub-kHz level on absorption line centers^1–4^. In order to bring such a comb revolution to the point of redefining spectroscopic databases such as HITRAN^5^, which are still mostly based on a pre-comb spectroscopy era, it is crucial to develop spectrometers that join an accurate frequency axis to a wide spectral coverage (>100 cm^−1^), which is the typical extension of absorption bands. This is easier to be performed in the near-infrared, thanks to the availability of commercial frequency combs and of a variety of widely tunable diode laser-based solutions. In this respect, demonstrations of accurate broad line surveys have been given for acetylene, ammonia and water in a sub-Doppler regime^6–10^ and more recently for carbon monoxide^11^ or carbon dioxide^12^ in a Doppler broadening regime.

In the mid-infrared (mid-IR) region, the development of such spectrometer is more challenging. A first requirement is the comb-referencing of the mid-IR probe laser: this has been obtained by a variety of approaches, such as down-conversion of the frequency comb to the mid-IR through difference frequency generation (DFG)^13,14^ or optical parametric oscillation (OPO)^15^, up-conversion of the probe laser to the near-IR through sum-frequency or second-harmonic generation (SFG/SHG)^16–19^, as well as referencing schemes applied to DFG- and OPO- based cw sources^4,20^. A second requirement is a widely tunable laser source. Up to a wavelength of 4.5 μm, a viable solution is represented by cw sources based on DFG or OPO processes in periodically-poled lithium niobate crystals: these have been exploited for sub-Doppler surveys over more than 50 cm^−1^ on CH_4_ lines near 3 μm and N_2_O lines near 4.5 μm^21^. Distributed-feedback quantum cascade lasers (QCLs) are a valuable alternative, but only over a narrower spectral range, as demonstrated by Galli *et al*.^22^ on CO_2_ lines near 4.3 μm. The widest spectral coverage achieved so far was obtained by a dual-comb approach^23^ that affords multi-parallel detection and extremely fast acquisition time. However, this comes at the price of an accuracy limited to ~300 kHz and of a setup composed of a pair of Hz-level-locked combs that can hardly be scaled for operation beyond 4.5 μm.

An extremely powerful alternative is represented by external cavity quantum cascade lasers (EC-QCLs): these enable single mode emission and frequency tuning in the mid-IR (from 4 to 12 μm) over ranges in excess of 100 cm^−1^, with a 100 mW optical power. Their adoption for precision spectroscopy has been hampered so far by a large amount of frequency noise, resulting in an optical linewidth of ~15 MHz over 50 ms^24^. This is one of the reasons why neither their frequency nor their phase has been so far locked to a frequency comb. Their use in combination with frequency combs has been demonstrated by the group of Newbury in an open loop regime^25^, which exploited the inherently fast and wide mode-hop-free tunability of these lasers, yet this approach could not reach an accuracy better than 800 kHz.

In this paper, we report for the first time frequency locking of an EC-QCL to a near-IR frequency comb, the former tuned from 7.6 to 8 μm, the latter at 1.9 μm from a Tm:fiber oscillator. The locking is obtained by slow feedback to the EC-QCL piezo with a 100 Hz servo bandwidth, which results in a 100 kHz frequency stability over 100 ms. In these conditions, N_2_O absorption spectra can be acquired and fitted with an overall uncertainty of about 63 kHz on the line center frequency. The addition of a fast feedback loop acting on an external acousto-optic frequency shifter is also discussed: this allows a narrowing of the laser emission line by a factor of 8, but it introduces a severe laser intensity noise that makes this choice counterproductive for the spectrometer performance.

## Experimental Setup

**Figure 1:**
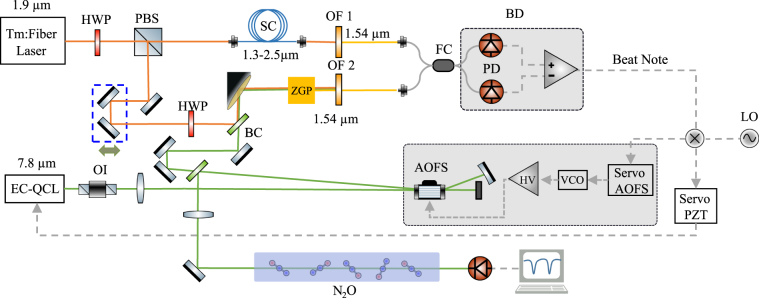
Experimental setup. Green line: EC-QCL optical path. Orange line: Tm-comb optical path. Grey dashed lines: electrical links. PBS: Polarizing beam splitter. PD: photodetector, PZT: Piezo actuator. OI: optical isolator. SC: supercontinuum. ZGP: Zinc-Germanium Phosphide. HWP: half wave plate. BC: beam combiner. OF: optical fiber coupler. BD: balance detector. LO: local oscillator. AOFS: acousto-optic frequency shifter. HV: high voltage amplifier. VCO: voltage controlled oscillator.

The layout of the spectrometer is sketched in Fig. 1. The near-IR frequency comb is based on an amplified Tm-fiber oscillator at 100 MHz delivering up to 1.5 W at 1.9 μm^26^. The EC-QCL (from Daylight Solutions, MHF series) operates at room temperature and provides single-mode emission in the 7.55–8.2 μm range with an output power up to 50 mW after optical isolation. The referencing scheme relies on an SFG process^27^, where part of the Tm output (100 mW) and of the EC-QCL (16 mW) are collinearly combined and focused by an off-axis parabolic mirror into an 8 mm long Zinc-Germanium Phosphide (ZGP) crystal. This generates a new comb at around 1.54 μm, hereafter called SFG comb. The frequency of the SFG comb is offset from the original comb by the EC-QCL frequency. By heterodyning the SFG comb against a spectrally broadened replica of the near-IR comb, a radio-frequency (RF) beat note (f_beat_) is eventually extracted, f_beat_ = |f_QCL_ − m∙f_rep_|, which allows the EC-QCL frequency (f_QCL_) to be determined against an integer multiple of the comb repetition frequency (f_rep_). Differently from^25^, where f_beat_ was tracked in real time by fast digitization followed by fast Fourier transform, f_beat_ was here steadily locked to a local RF oscillator.

Two locking schemes have been implemented and tested. A first scheme makes use of one servo only, providing feedback to the EC-QCL through the available piezo modulation port. Due to a bandwidth limit of 100 Hz, it was not possible by this approach to go beyond frequency locking, essentially compensating for the laser frequency drift. In a second scheme, to achieve a faster frequency correction and to explore the feasibility of phase locking, we added a second servo acting on an acousto-optic frequency shifter (AOFS). We recurred to external frequency actuation rather than to the laser current control because for our laser the current tuning was high-passed at 10 kHz by the manufacturer, thus inhibiting any laser frequency control in the 100 Hz-10 kHz range. As sketched in Fig. 1, the SFG branch of the setup is aligned to the beam diffracted by the AOFS. This was arranged in a double-pass configuration to benefit from doubled frequency shifts while suppressing misalignments due to the changing diffraction angle.

## Results and Discussion

**Figure 2:**
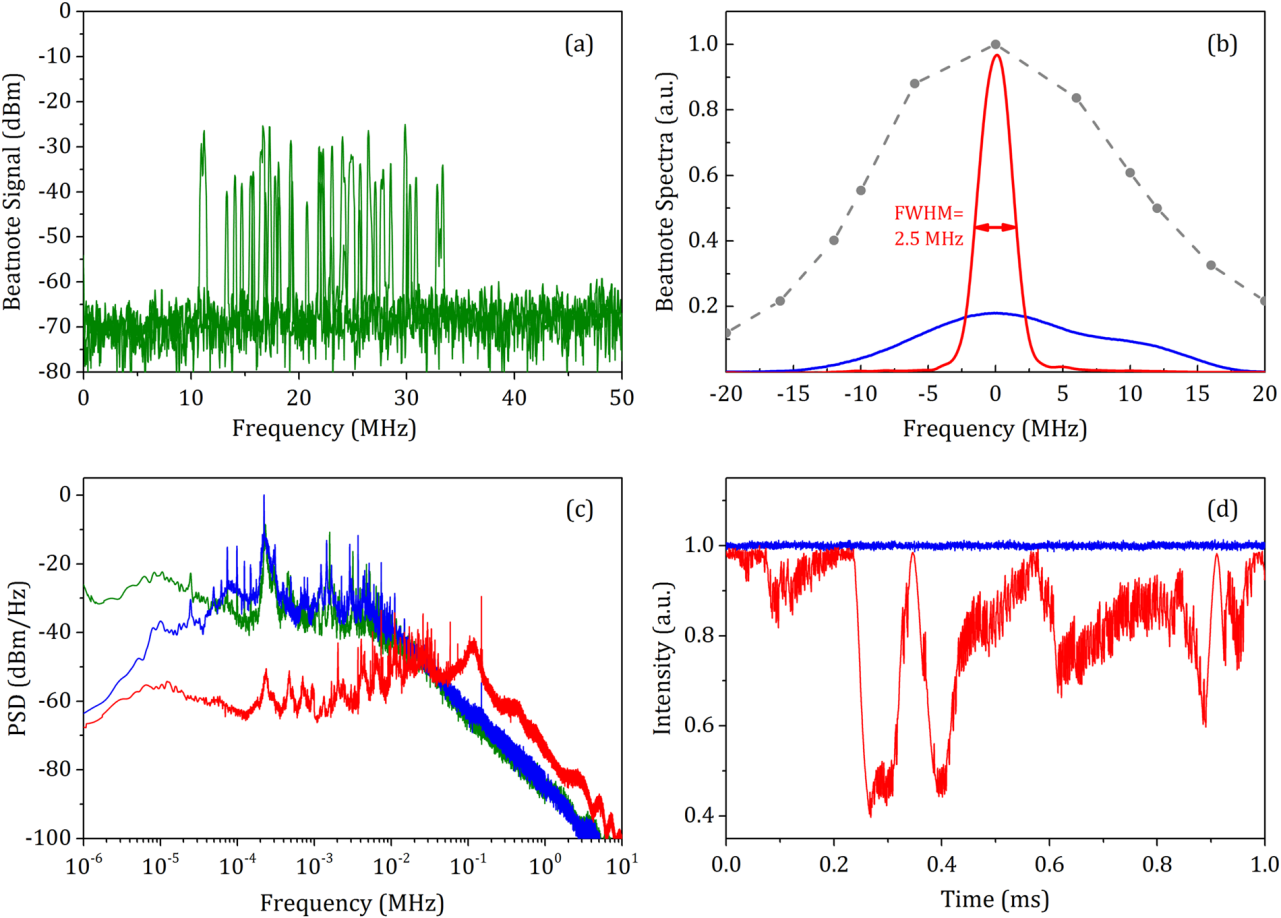
Color code: green (free running), blue (slow locking), red (fast locking). (**a**) Beat note signal spectrum acquired with a sweep time of 6 ms at a 50 kHz resolution bandwidth, showing a nearly 20 MHz large laser jittering window at a ms time scale. (**b**) Averaged electrical spectrum of the beat note signal under slow and fast locking, as compared to the diffraction efficiency response of the AOFS (grey dashed dotted line). (**c**) Power spectral density of the error signal. (**d**) Scope traces of the laser intensity in locking condition.

Figure 2(a) reports the beat note spectrum in a free-running regime acquired with a sweep time of 6 ms at a 50 kHz resolution bandwidth. It can be noticed that the EC-QCL frequency experiences a jitter of about 20 MHz at the ms timescale which is comparable with the value reported by the Newbury group in the unique frequency characterization of an EC-QCL present in the literature^24^. The satisfactorily high signal-to-noise ratio (SNR) of more than 30 dB derives from an efficient nonlinear interaction in ZGP, which leads to an SFG comb power of ~80 nW, and from the use of balanced detection, which gives a reduced intensity noise floor and a 3 dB higher SNR as compared to direct detection. Figure 2(b) reports the averaged profiles of the beat note under slow (blue) and fast (red) locking. In the slow case, the lock compensates for the frequency drifts of the laser and maintains its average frequency at a predetermined offset from the comb mode pattern. On the other hand, it is not able to provide an appreciable line narrowing since the laser is affected by large frequency excursions beyond 100 Hz, thus beyond the available control bandwidth. This leads to an effective linewidth of about 21 MHz, slightly worse than the 15 MHz value observed in^24^ for a 4.5 μm EC-QCL. The addition of a second faster feedback loop better compensates for the laser frequency jitter and narrows the emission line down to 2.5 MHz (Fig. 2(b)), which corresponds to an improvement by a factor of 8 in the available spectral resolution once the laser is applied to spectroscopy. The electrical spectra of the error signal reported in Fig. 2(c) for the free-running and locking regimes show that the second loop provides an efficient noise suppression up to about 30 kHz. Extending further the control bandwidth resulted in an unstable behaviour due to the rather high 1.6 μs delay introduced by the AOFS, which is responsible for the servo-bump at 100 kHz and for the excess noise beyond it. Over a measurement time of 100 ms, the counted beat note suffers from an rms fluctuation of 100 and 35 kHz for the slow and fast lock, respectively.

The higher frequency stability and spectral resolution afforded by the fast locking were found to be traded off by a severely degraded intensity noise. The oscilloscope traces reported in Fig. 2(d) show that the intensity drops by more than 50%. These oscillations emerge because the frequency jitter of the laser forces the AOFS to work outside its modulation bandwidth, i.e., at frequencies where its diffraction efficiency is degraded. This is better quantified in Fig. 2(b) by the comparison between the beat note spectrum (blue curve) and the diffraction response of the AOFS (dashed grey curve), which have comparable widths. It is worth noting that the intensity noise deriving from such an issue also impacts the quality of the feedback locking loop and could not be trivially solved by adoption of a faster AOFS.

To benefit from a constant power level (blue trace in Fig. 2(d)), spectroscopy measurements were performed with the EC-QCL slowly locked to the comb, under two different frequency scanning strategies. The first one exploits the application of a step-wise modulation to the EC-QCL piezo, forcing the laser frequency to jump from one comb mode to the next one, which implies unlocking and relocking at every spectral step. The procedure is robust due to the capture range of more than 20 MHz afforded by the large laser linewidth, which could easily accommodate piezo nonlinearities. The voltage/frequency steps result in an evenly spaced frequency axis with spectral points at every f_rep_ (100 MHz in our case). This approach can be applied for spectral scans up to 0.9 cm^−1^, which is the limit given by the piezo. However, this methodology can be easily extended to tens of wavenumbers with a remote control system that takes charge of driving both piezo and rotation stage of the laser. To achieve a denser sampling of narrow spectral features, we tested both an interleaving of spectra acquired with different comb repetition rates and, as a second frequency scanning strategy, the tuning of the rep-rate while keeping a steady lock between the EC-QCL and the comb.

**Figure 3:**
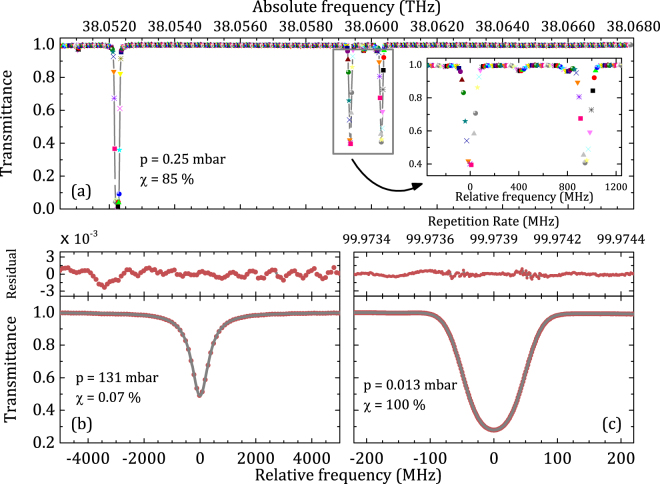
(**a**) Absorption spectrum at 298 K of a 85% air-diluted N_2_O sample at a pressure of 0.25 mbar near 1269 cm^−1^, with a comb-defined frequency axis. Inset: zoomed-in view of the P(25) doublet, with interleaved spectra for a denser spectral sampling. (**b**) Absorption spectrum of the P(18) line of N_2_O with a 0.07% dilution at 131 mbar with a 100-MHz frequency grid (bottom panel) and residuals from a Voigt fitting (top panel). (**c**) Absorption spectrum of the same line in a pure sample at 0.013 mbar (bottom panel), and residuals from a Gaussian fitting (top panel). The spectrum is in this case acquired by a 4 Hz stepping of the comb repetition frequency (1.5 MHz spaced optical frequency grid). In the figure, χ is the dilution factor.

Figure 3(a) reports an example of absorption spectrum near 1269 cm^−1^ of an 85%-air-diluted N_2_O sample housed in a 66 cm long optical cell at a pressure of 0.25 mbar. The spectrum extends over 0.6 cm^−1^ and presents 12.5 MHz-spaced points due to the interleaving of eight scans acquired at slightly detuned repetition frequencies (by ~30 Hz). The inset provides a zoomed-in view of the P(25) doublet and better highlights repeatability and absolute positioning of spectral points.

A quantitative analysis has been performed for the spectra reported in Fig. 3(b) and (c), both referring to the P(18) N_2_O line of the 1000-0000 band. In the first case, thanks to a 0.07%-diluted sample at a relatively high pressure of 130 mbar, a 100MHz sampling is sufficiently dense to reproduce the spectral shape and enable a reliable fitting with a Voigt profile. On a statistical ensemble of 100 acquisitions, the fitting provides an rms deviation of 500 kHz for the line-centre frequency, which is equivalent to 7 parts over 10^4^ with respect to a 750 MHz linewidth. The uncertainty primarily reflects the SNR of the measurement, which amounts to ~1000 for the single spectrum. Apart from the interference of a weak neighbouring line and from the presence of etalon effects at the 10^−3^ level, the residuals from the fitting do not show, at such a level of SNR, any appreciable departure from the Voigt profile. A more stringent test on precision (Fig. 3(c)) was obtained on the same line at a pressure of 0.013 mbar, i.e. in conditions where the collisional broadening is negligible and the absorption linewidth is Doppler dominated to an estimated value of 70.5 MHz. A 1.5 MHz spectral sampling is ensured by a 4 Hz stepping of the comb rep-rate. This occurs at every 100 ms so that a 540 MHz large spectrum is acquired in 36 s. The statistical uncertainty on the line-centre frequency was inferred by repeating several times the measurement of the same absorption spectrum; the line centres retrieved from the fitting of those spectra are described by a Gaussian distribution with a rms deviation of 47 kHz, which implies that when 10 spectra are averaged together (as it shown in Fig. 3(c)), the resulting statistical uncertainty can be safely estimated at the 15 kHz level. Despite the relatively fast scanning speed of the rep-rate, no appreciable bias from the frequency comb rep-rate scanning direction was found. The averaged residuals of 10 consecutive spectra fitted with a Gaussian profile (Fig. 3(c)) reveal an SNR of 900. Apart from small etalons whose contribution is below the 10^−3^ level, the residuals are almost flat within the SNR of the measurement.

In terms of systematic uncertainty, several sources have been identified and quantified. The stability of the Global-Positioning-System-based frequency standard (5 parts over 10^12^ at 36 s), which serves as a reference for the comb and for the frequency offset between comb and EC-QCL, translates into a frequency error of ~0.5 kHz. The uncertainty of the absolute pressure gauge used in the experiments (10% at the pressure of 0.013 mbar) provides contribution of less than 0.5 kHz. The cell leakage, of the order of 0.0065 mbar/h, gives a sub-kHz impact over a typical measurement time of 6 minutes corresponding to an averaging of 10 spectra. The extrapolation of line centre frequencies to zero pressure through the air pressure-induced shift coefficients instead of selfpressure-induced shift coefficients^28^ does not impact the accuracy by more than 2 kHz at the very low pressure adopted herein. Baseline distortions occurring due to etalons and laser power drifts have been found to give a higher contribution, up to 10 kHz at the edges of the tuning range, but the major systematic limitation derives from an asymmetrical jittering of the laser around the local oscillator frequency, which leads to a beat note barycentre slightly detuned from the local oscillator (see Fig. 2(b)). Such detuning has been accounted for by registering the laser lineshape, as well as its absolute position from the nearest comb mode, through the electrical spectrum of the beat note between the EC-QCL and the comb during the spectral scans, then deconvolving each absorption profile by the beat note itself. As the beat note could be measured with a very high SNR (>10^4^) over the 6 minutes-long measurement time, the remaining uncertainty relates to the calibration of the frequency responses of the detector and of the RF filters and amplifiers used to detect the beat note itself. This has been estimated at a level of 60 kHz by comparing the centre of the same absorption line measured under different conditions, either reversing the sign of the lock or changing the local oscillator frequency. It is worth noting that the impact of an asymmetric jittering behaviour is not appreciable from the residuals (Fig. 3(c)). The total systematic (Type B) uncertainty amounts to about 61 kHz and gives rise to an overall uncertainty of 63 kHz by quadrature addition with a statistical (Type A) contribution of 15 kHz (10 averaged spectra). The resulting line centre-frequency of the P(18) line is 38052238584(63) kHz; the HITRAN value for the line-centre frequency is only 2.7 MHz above our determination while the HITRAN value has relatively large nominal uncertainty range of 3–30 MHz. The Doppler width retrieved from spectral fitting is equal to 73.9 MHz, thus 2.4 MHz higher than the expected value, but this precisely reflects the instrumental broadening (21 MHz) of our laser, once the typical quadrature addition law for Gaussian widths is applied.

**Figure 4:**
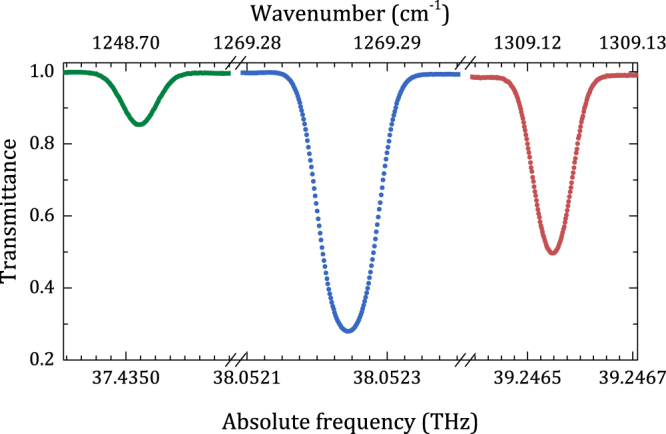
N_2_O spectroscopy at 0.013 mbar over 65 cm^−1^. Green line: P(40), 37435005.26(10) MHz; blue line: P(18), 38052238.584(63) MHz; red line: R(31), 39268217.812(78) MHz.

The spectrometer is able to fully exploit the emission range of the EC-QCL, which extends over more than 100 cm^−1^. A demonstration of such large tunability is given in Fig. 4, which reports spectroscopy of lines in the P and R branches of N_2_O, namely P(40) and R(31), lying 65 cm^−1^ far apart. The lines have been measured by a remote control system that allows the uploading of the desired line list from HITRAN, the tuning of the EC-QCL to the target frequencies by an active loop fed by the reading of a wavemeter, the locking of the laser to the comb, the scanning of the comb rep-rate and the absorption line acquisition, finally the laser unlocking and the tuning to the next line frequency. Interestingly, the rather large jittering of the laser facilitates the relocking procedure, allowing tens of lines to be repeatedly and robustly acquired. Measurements are ongoing on the P and R branches of N_2_O to infer the rotational constants of ground and excited state. For such a broad survey, statistical uncertainties are expected to be averaged out consistently. For collisional studies, instead, where molecular line shapes need to be discriminated well below the 10^−3^ level, a hurdle is represented by the need to deconvolve the absorption spectra with a significantly large laser emission. In such respect, narrower DFB-QCLs or a new-generation of EC-QCLs phase locked to the comb represent a more adequate solution. In terms of applicability of the proposed scheme to other wavelengths it is worth to note that the spectrometer range could be easily extended up to 12 μm simply by changing the laser head. It is even possible to replace the Tm:comb with a more common Er:comb, by exploiting for the SFG process either the long-wavelength part of its supercontinuum or its main output at 1.55 μm, in the former case with the same crystal used here (ZGP or also AgGaSe_2_), in the latter case recurring to AgGaS_2_ or orientation patterned GaP.

## Conclusions

We have reported on the architecture and performance of a comb-referenced spectrometer that provides frequency uncertainty of ~63 kHz and tunability over 100 cm^−1^ near 7.8 μm. The spectrometer lends itself to broad line surveys and to the redefinition of HITRAN data in a region where previous experiments primarily relied on Fourier transform infrared spectroscopy. An accurate survey of P and R branches of the fundamental 1000-0000 band of N_2_O is ongoing, and other molecules, such as H_2_O_2_ and CH_4_, are likely to be targeted in the currently available spectral region.
