# Author Correction: Absolute spectroscopy near 7.8 μm with a comb-locked extended-cavity quantum-cascade-laser

**DOI:** 10.1038/s41598-020-69879-y

**Published:** 2020-08-07

**Authors:** Marco Lamperti, Bidoor AlSaif, Davide Gatti, Martin Fermann, Paolo Laporta, Aamir Farooq, Marco Marangoni

**Affiliations:** 1 Physics Department of Politecnico di Milano and IFN-CNR, Via G. Previati 1/C, 23900 Lecco, Italy; 2 King Abdullah University of Science and Technology (KAUST), Clean Combustion Research Center (CCRC), Thuwal, 23955 Saudi Arabia; 3 IMRA America Inc., 1044 Woodridge Avenue, Ann Arbor, Michigan 48105 USA

Correction to:* Scientific Reports*10.1038/s41598-018-19188-2, published online 22 January 2018

This Article contains errors.

The legend of Figure 4 is incorrect:

“N_2_O spectroscopy at 0.013 mbar over 65 cm^−1^. Green line: P(40), 37435005.26(10) MHz; blue line: P(18), 38052238.584(63) MHz; red line: R(31), 39268217.812(78) MHz.”

should read:

“N_2_O spectroscopy at 0.013 mbar over 65 cm^−1^. P(40), 37435005.58(10) MHz; blue line: P(18), 38052238.986(63) MHz; red line: R(31), 39268218.298(78) MHz”

As a result, there is an error in the Results and Discussion section where,

“The resulting line centre-frequency of the P(18) line is 38052238584(63) kHz; the HITRAN value for the line-centre frequency is only 2.7 MHz above our determination while the HITRAN value has relatively large nominal uncertainty range of 3–30 MHz”

should read:

“The resulting line centre-frequency of the P(18) line is 38052238986(63) kHz; the HITRAN value for the line-centre frequency is only 2.7 MHz above our determination while the HITRAN value has relatively large nominal uncertainty range of 3–30 MHz.”

